# Context dependent differences in working memory related brain activity in heavy cannabis users

**DOI:** 10.1007/s00213-021-05956-y

**Published:** 2021-08-27

**Authors:** Emese Kroon, Lauren Kuhns, Janna Cousijn

**Affiliations:** 1 Neuroscience of Addiction (NofA) Lab, Department of Psychology, University of Amsterdam, P.O. box 15916, 1001 NK Amsterdam, Netherlands; 2 The Amsterdam Brain and Cognition Center (ABC), University of Amsterdam, Amsterdam, Netherlands; 3 Department of Psychology, Education & Child Studies, Erasmus University Rotterdam, Rotterdam, Netherlands

**Keywords:** Cannabis, Cognitive control, Context, fMRI, Working memory

## Abstract

**Rationale:**

Compromised cognitive control in cannabis use–tempting situations is thought to play a key role in the development of cannabis use disorders. However, little is known about how exposure to cannabis cues and contexts may influence cognitive control and the underlying neural mechanisms in cannabis users.

**Objectives:**

Working memory (WM) is an attention reliant executive function central to cognitive control. In this study, we investigated how distracting cannabis words affected WM load–dependent performance and related brain activity in near-daily cannabis users (*N* = 36) relative to controls (*N* = 33).

**Methods:**

Brain activity was recorded during a novel N-back flanker WM task with neutral and cannabis flankers added as task-irrelevant distractors.

**Results:**

On a behavioural level, WM performance did not differ between groups, and the presence of cannabis flankers did not affect performance. However, in cannabis users compared to controls, the presence of cannabis flankers reduced WM load–related activity in multiple regions, including the insula, thalamus, superior parietal lobe and supramarginal gyrus.

**Conclusions:**

The group specificity of these effects suggest that cannabis users might differ from controls in the way they process cannabis-related cues and that cannabis cue exposure could interfere with other cognitive processes under cognitively demanding circumstances. Future studies should focus on the role of context in cognitive control–related processes like WM and attention to further elucidate potential cognitive impairments in heavy cannabis users and how these relate to loss of control over drug seeking itself.

**Supplementary Information:**

The online version contains supplementary material available at 10.1007/s00213-021-05956-y.

## Introduction

Cognitive control deficits play an important role in substance use disorders (SUDs), including cannabis use disorders (CUDs); the inability to refrain from cannabis use in tempting and arousing cannabis use–related context is thought to support the development and maintenance of CUD (Goldstein and Volkow 2011). Several studies indicated compromised cognitive control (Cousijn et al. 2013b; Charles-Walsh et al. 2016), hyperresponsivity to cannabis-related cues (e.g. Cousijn et al. 2013a; Zhou et al. 2019) and altered functioning of the underlying brain areas (Kober et al. 2014) in cannabis users; however, relatively little is known about how these processes interact. The goal of this study was to investigate the influence of a distracting cannabis use–related context on cognitive control in cannabis users.

Working memory (WM) is considered to be a central aspect of cognitive control and is essential for many higher-order cognitive processes (Unsworth and Engle 2007). WM requires attention and involves the ‘online’ maintenance and manipulation of information. Multiple types of WM tasks have shown robust activation in a widespread network of frontoparietal brain areas (Linden et al. 2003; Owen et al. 2005). While several studies have shown that cannabis intoxication and heavy cannabis use can impair WM performance, these impairments are not consistently found (Schoeler and Bhattacharyya 2013; Bossong et al. 2014). Several functional magnetic resonance imaging (fMRI) studies have examined the relationship between heavy cannabis use and brain activity and connectivity during WM tasks. Although group differences in performance are rarely found, there is some evidence for differences in brain activity and WM network functioning (including primarily frontal and parietal regions; Owen et al. 2005). Multiple studies have found that, compared to controls, heavy cannabis users show increased activity in WM-related areas (e.g. prefrontal cortex) and recruit additional areas that are not usually expected to play a crucial role in WM (e.g. subcortical areas involved in emotion and reward processes), without differences in WM performance (Kanayama et al. 2004; Smith et al. 2010). This over-recruitment is often interpreted as a compensation strategy needed in order to perform on a behaviourally similar level as controls (Bossong et al. 2014) and might be more prominent in early-onset cannabis users (Becker et al. 2010). With regard to WM network functionality, stronger network response during an N-back WM task is associated with an increase in cannabis use 6 months later (Cousijn et al. 2014b), suggesting that individuals who require more network effort for accurate performance are more likely to escalate cannabis use over the following 6 months.

Based on most addiction theories (e.g. Robinson and Berridge 1993; Koob and Volkow 2010), strong fronto-limbic reward and emotion-related reactivity in response to cannabis cues and contexts in fronto-limbic brain areas would interfere with frontoparietal cognitive control–related processes, biasing cognition towards cannabis use (e.g. craving, attentional bias, approach actions). Therefore, on a conceptual level, WM performance in tempting and challenging cannabis-related contexts may more closely relate to actual use and CUD severity than WM performance in non-tempting neutral context. If this is the case, some specific cannabis-related deficits in WM may have been overlooked in previous studies using relatively neutral WM tasks. Indeed, multiple studies have shown that context-dependent emotional state affects performance as well as brain activity during cognitive control tasks (Erk et al. 2007; Iordan et al. 2013). For example, cannabis users show lower inhibition than control participants when the task requires inhibiting risky responses in the foresight of a potential reward, but not in a more classic rule-based task with inhibitory responses based on neutral stimuli (Griffith-Lendering et al. 2012). Similarly, weekly cannabis users performed worse than non-using controls on an adapted Stroop task including cannabis-related words, while performing similarly to controls when presented with neutral words (Cousijn et al. 2013b). This increased attentional bias for cannabis-related words was associated with severity of dependence (Field 2005; Cousijn et al. 2013b). Aside from strong behavioural reactivity to cannabis-related cues, cannabis users also displayed relatively higher activity in reward-related limbic regions compared to controls when presented with cannabis cues (e.g. Cousijn et al. 2013a; Zhou et al. 2019). These findings support the idea that differences between cannabis users and non-users in attention reliant cognitive processes like WM may be more evident in a cannabis-related context than in a cannabis-unrelated or neutral context; however, research into this area is currently missing.

In this study, we aimed to investigate the influence of a distracting cannabis use–related context on WM load–dependent performance and brain activity during a WM task in heavy cannabis users relative to controls. We developed an N-back flanker task in which cannabis and neutral words flanked the standard letter N-back task (Mackworth 1959). Previously, flankers have been used in a variety of cognitive tasks to induce a task-irrelevant component that distracts from the main goal of the task (e.g. Mclean et al. 2014; Trujillo et al. 2021). The cannabis-related words used in the current study have previously been shown to induce attentional bias in heavy cannabis users, interfering with the colour naming of cannabis relative to the neutral words in a Stroop task (Cousijn et al. 2013b). While the flanker condition increases attentional task load, requiring participants to actively inhibit the flankers, the cannabis-related words add an additional attentional component for cannabis users specifically. Similar to previous studies with a standard N-back task, we expected performance to be WM load dependent in both groups with lower accuracy and longer reaction times for high WM load (2-back trials) than for low WM load (1-back trials). However, we expected cannabis flankers to increase task load (i.e. effort) in cannabis users only, such that performance would be lower but WM-related frontoparietal brain activity would be higher in cannabis users compared to controls for cannabis flanker trials, but not for neutral flanker trials. To further explore the potential mechanisms underlying group differences in brain activity, we investigated whether individual’s peak activity in significant clusters covaried with WM load–dependent performance and severity of cannabis use.

## Materials and methods

The current study was part of a larger project that aimed to investigate neurocognitive processes involved in heavy cannabis use and CUD and will only describe the results of the participants that completed the N-back flanker task. The ethical committee of the department of psychology of the University of Amsterdam approved the study (2015-DP-6387), and all participants were fully informed and provided informed consent before participation. All participants received monetary compensation for their participation.

### Participants

A total of 38 heavy cannabis users and 34 healthy controls between 18 and 25 years old were recruited through online (e.g. social media) and offline (e.g. cannabis outlets) advertisements in the Amsterdam area. Potential participants were screened during a telephone interview before inclusion. Heavy cannabis users were required to use cannabis 10–30 times a month for at least 2 years, while control participants used cannabis at least once, but no more than 50 times during their life and not during the last year. General exclusion criteria were excessive alcohol use (Alcohol Use Disorder Identification Task (AUDIT) score > 12; Saunders et al. 1993; Källmén et al. 2019), smoking more than 20 cigarettes per day, the current use of prescription or illicit psychoactive drugs other than cannabis, substance use other than cannabis over a hundred times, previous or current serious physical (requiring regular visits to a specialists) or mental health (major axis-1 disorders) problems, leaving school before age 16 and previous or current treatment for CUD or plans to enter treatment. Groups were closely matched on age, sex, IQ, educational level, alcohol use, smoking, substance use other than cannabis and mental health outcomes (Table 1).

**Table 1:** Sample characteristics

Measures	Cannabis group	Control group
***N*** (% male)	36 (53)	33 (49)
Age, median	21	21
Estimated intelligence, WAIS-IV matrix reasoning and similarities mean (SD)	21.03 (4.16)	22.00 (4.37)
Educational level, highest completed education, median	2	2
Impulsivity (BIS-11), mean (SD)	70.86 (6.89)	71.35 (5.70)
ADHD (CAARS), median	16	15
Depression (BDI), median	4	2
Trait Anxiety (STAI-Trait), median	33.5	34
State Anxiety (STAI-State), median	29.5	31
Alcohol use and related problems (AUDIT), median	6	5
Cigarette smoking, % cigarette smokers	47	42
Cigarettes per day (cigarette smoking), mean (SD)	9.74 (4.19)	9.75 (5.56)
Nicotine dependence (FTND), mean (SD)	2.88 (1.96)	2.29 (1.64)
Lifetime other drug use, median	12^*^	0^*^
Cannabis use and related problems (CUDIT-R), median	13 ^*^	0^*^
Cannabis use onset (age), mean (SD)	15.39 (1.92)	-
Cannabis use onset heavy use (age), mean (SD)	17.63 (1.96)	-
Cannabis gram per week, mean (SD)	2.74 (2.31)	-
Cannabis use days per week, mean (SD)	4.88 (1.67)	-
Cannabis use disorder (SCID DSM-5), mean (SD)	3.50 (1.63)	-
Self-reported cannabis abstinence (days), mean (SD)	1.28 (0.91)	-

^*^p < 0.001 for group comparison; medians are reported in case of non-parametric assessment of group differences and for assessment of group differences based on count data with over 2 categories; *SD*, standard deviation; *WAIS-IV*, Wechsler Adult Intelligence Scale IV (Wechsler 2012); *CAARS*, Conners’ Adult ADHD Rating Scales (Sandra Kooij et al. 2008); *BDI*, Beck Depression Inventory (Beck et al. 1961); *STAI*, State Trait Anxiety Inventory (Spielberger and Sydeman 1994); *AUDIT*, Alcohol Use Disorder Identification Test (Saunders et al. 1993); *CUDIT-R*, Cannabis Use Disorder Identification Test (Adamson et al. 2010); *FTND*, Fagerström Test for Nicotine Dependence (Heatherton et al. 1991); *SCID-5*, Structured clinical interview for DSM-5—Cannabis use disorder symptoms (First 2015).

Participants were instructed to refrain from using alcohol and drugs (except for nicotine and caffeine) 24 h before the test session (see Table 1 for self-reported cannabis abstinence). During the test session, a urine drug test was performed to identify recent use of amphetamine, methamphetamine, benzodiazepines, cocaine, opiates and cannabis (THC). Participants that tested positive (except for THC in the heavy cannabis use group) were excluded from the analysis.

### Questionnaires

Cannabis use and related problems during the last 6 months were assessed using the Cannabis Use Disorder Identification test (CUDIT-R; Scores > 12 indicative of potential CUD; Adamson et al. 2010). Similarly, the AUDIT (Saunders et al. 1993) and Fagerström Test for Nicotine Dependence (FTND; Heatherton et al. 1991) were used to identify last 6 months of alcohol and cigarette use and related problems, respectively. A substance use history questionnaire was used to assess frequency, quantity and onset of alcohol use, cigarette use, cannabis use as well as other illicit drug use. Additionally, a DSM-5 structured clinical interview for cannabis dependence (SCID DSM-5 CUD; score 2–3 = mild, score 4–5 = moderate, score > 5 = severe; First 2015) was administered to assess cannabis dependence. Severity of depression (Beck’s Depression Inventory (BDI); Beck et al. 1961), anxiety (State-Trait Anxiety Inventory (STAI) Spielberger and Sydeman 1994) and ADHD ( Conners’ Adult ADHD rating Scales (CAARS); Sandra Kooij et al. 2008) symptoms were assessed. Additionally, intelligence was estimated using the matrix reasoning and similarities subtests of the Wechsler Adult Intelligence Scale IV (WAIS-IV; Wechsler 2012) and educational level classified with a single question assessing highest completed education (Dutch higher education levels; 1 = MBO (vocational education) or less, 2 = HBO (university of applied sciences), 3 = WO (university)).

### N-back flanker task

**Fig. 1:**
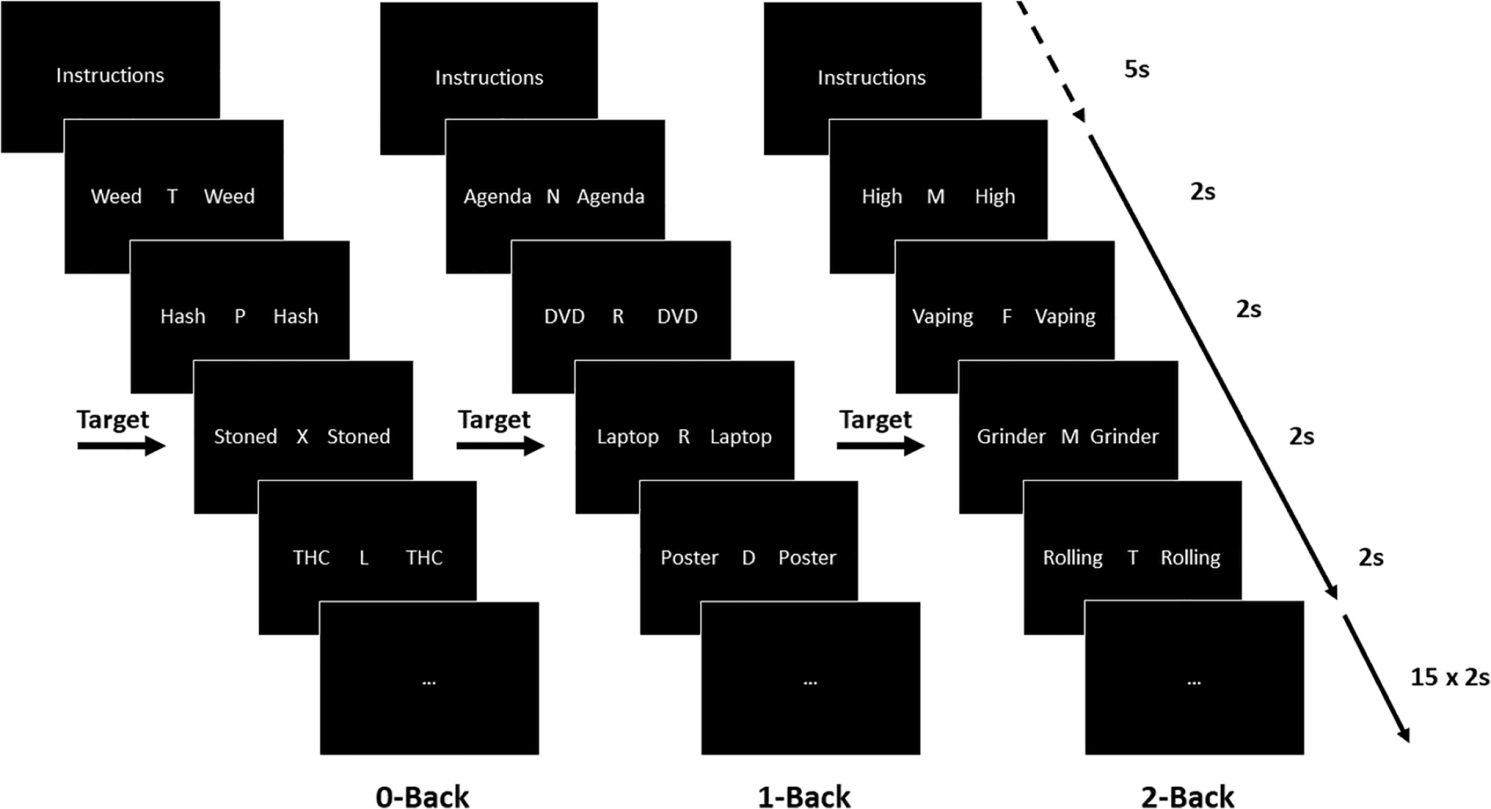
Task overview. Stimuli were similar to regular letter N-back stimuli, presenting a letter in the centre of the screen during every trial. Cannabis (see 0-back and 2-back) or neutral (see 1-back) words were simultaneously presented on both sides of this letter during the entire trial. Letters and words changed every trial, but the flanking words (cannabis or neutral) were consistent over each block of 15 trials. Before the start of the task, participants were given sufficient time to read instructions for the difference trial types. Block-specific instructions were presented again for 5 s at the start of each block, followed by a block of 15 trials lasting 2 s each resulting in a total block length of 30 s

The participants performed the adapted N-back flanker task, developed using E-prime 2.0 software (Psychology Software Tools Schneider et al. 2002), while fMRI blood-oxygen-level–dependent (BOLD) signals were recorded. The task included 6 different types of blocks including three WM loads (0-back (recognition), 1-back (low WM load) and 2-back (high WM load)) and two flanker types (neutral or cannabis; 3 × 2 factorial design). All block types were presented twice in a fixed order resulting in a total of 12 blocks of 15 trials. Each trial was presented for a fixed duration of 2 s, resulting in 30 s per block and a total task length of 7 min (including the 5-s instructions before the start of each block; Fig. 1). During each trial, a capital letter was presented with either a neutral or cannabis ‘flanker’ on the left and right side. Flankers were either cannabis-related words (cannabis-context trials; e.g. ‘joint’ or ‘high’) or neutral stationary words (neutral-context trials; e.g. ‘paperclip’ or ‘printer’) and were matched on word length and number of syllabi. Substance-related words and matched neutral words have been validated for use in designs assessing attentional bias in substance users (Ataya et al. 2012). The included neutral and cannabis words were previously used in an attentional bias study using the modified cannabis Stroop task (Cousijn et al. 2013b). The results of this study show that heavy cannabis users were slower naming the colour in which cannabis words were printed than they were at naming the colour in which neutral words were printed, indicating an attentional bias towards the cannabis relative to the neutral words. Before the 0-back (baseline recognition) blocks, participants were instructed to press the target button when a letter ‘X’ (the target) was presented. In the 1-back (low WM load) blocks, participants were instructed to press the target button when the presented letter was identical to the letter presented during the previous trial. Similarly, in the 2-back (high WM load) blocks, participants were instructed to press the target button when the presented letter was identical to the letter presented in the trial before the previous trial. During all non-target trials, participants pressed the non-target button. Each block of 15 trials included 5 target trials. No feedback on performance was provided during or at the end of the task.

### Procedure

The consent procedure was followed by a first series of pen-and-paper questionnaires and the WAIS subscale assessments. The urine drug test was performed before practising the scanner tasks. After MRI safety screening, participants completed a 50-min scan session. After scanning, two series of pen-and-paper questionnaires and additional behavioural tasks were conducted.

### Imaging parameters and preprocessing

A 3 T Intera MRI scanner (Philips Intera, Best, Netherlands) with a 32-channel SENSE head coil, located at the Spinoza Centre for Neuroimaging at the University Medical Center Amsterdam, was used for image acquisition. For each participant, a high-resolution structural scan was obtained for anatomical reference (T1 turbo field echo, TR = 8.2 s, TE = 3.8 ms, 220 slices, slice thickness = 1.0 mm, field of view (FOV) = 240 × 188 mm, voxel size = 1 × 1 mm, flip angle = 8°). BOLD responses were recorded during the N-back flanker task using a T2* single-shot echo-planar imaging (EPI) sequence (TR = 2.0 s, TE = 28 ms, 37 slices, slice thickness = 3 mm, inter slice gap = 0.3 mm, FOV = 240 × 240 mm, voxel size = 3 × 3 mm, flip angle = 76°).

Preprocessing was conducted with FSL FEAT (FMRIB’s Software Library version 5.0.6, part of fMRI Expert Analysis Tool version 6.0). Non-brain tissue and skull were removed using BET (Brain Extraction Tool) where after regular-up slice time correction, high-pass filtering (sigma = 90), motion correction (using MCFLIRT), spatial smoothing (5-mm full-width-half-maximum Gaussian kernel) and prewhitening were applied. The functional data was then registered to the participant’s structural T1-weighted image and transformed to standard space (MNI-152) using FNIRT (FMRIB’s non-linear registration tool).

### Data analysis

#### Behavioural data analyses

Sample characteristics were compared over groups using either independent sample *t*-tests, Mann–Whitney *U* tests (in case of violation of assumptions) or chi-square tests (in case of categorical data) in RStudio (version 1.1.463; R Core Team 2013). Trials without a response and those with a reaction time below 200 ms were excluded. Then, a linear mixed effects model approach with maximum likelihood estimation and stepwise model selection was used to assess whether WM load, flanker type, group or their interactions affected task performance measured as accuracy (percentage correct responses) and reaction time on accurate trials (RT). In all models, the intercept was allowed to vary over participants (random intercept) while random slopes were included for WM load and flanker type to account for repeated measures within participants. Model fit was assessed using Akaike’s information criterion (AIC) to compare models.

#### fMRI analyses

A check for excessive motion did not result in the exclusion of participants (max. motion = 2.36 mm). A general linear model (GLM, ordinary least squares) analysis was conducted using FSL’s FEAT. All 6 different trial types (WM load (3) × flanker type (2)) were added as regressors and convolved with a double gamma hemodynamic response function. Temporal derivatives were added to the model to improve fit. Three contrasts were created to assess the main effect of flanker (cannabis (c) > neutral (n)), the main effect of WM (2-back (2) > 1-back (1)) and their interaction ((2c > 1c) > (2n > 1n)). Next, whole-brain mixed effects (FLAME1) group analyses with cluster-wise correction for multiple comparisons (*Z* > 2.3, cluster-based significance *p* < 0.05) were conducted, where independent sample *t*-tests were used to assess group differences (control – cannabis) on each of the three contrasts.

For descriptive purposes, we identified regions of maximal effect within the identified cluster by thresholding the contrast maps at *Z* > 3.1 (> 10 voxels per region) and extracted mean peak activation for each individual within these regions using FSL Featquery. This allowed for exploratory inspection of the direction of the effects and how individual mean peak activation levels within these specific regions covary with heaviness of cannabis use (gram per week) and severity of cannabis use–related problems (CUDIT-R; SCID DSM-5 CUD) within the group of cannabis users. Additional exploratory regression analyses were conducted to assess whether task performance was predictive of individual mean peak activation.

## Results

### Sample characteristics

Three participants were excluded for testing positive on a drug other than cannabis (1 cannabis group, 1 control group) during the test session or for not following task instructions (1 cannabis group). The final sample consisted of 36 heavy cannabis users and 33 controls. As can be seen in Table 1, groups did not differ on sex (*χ*^*2*^(1, *N* = 69) = 0.13, *p* = 0.72), age (*Z* =  − 0.03, *p* = 0.78), estimated IQ (*t*(65) = 0.95, *p* = 0.35), educational level (*χ*^*2*^(2, *N* = 69) = 4.86, *p* = 0.09), impulsivity (*t*(66) = 0.33, *p* = 0.74), ADHD symptoms (*Z* = -0.57, *p* = 0.57; CAARS), depression (*Z* =  − 1.94, *p* = 0.052; BDI) and trait (*Z* =  − 0.70, *p* = 0.49) nor state (*Z* =  − 0.59, *p* = 0.56) anxiety (STAI). With regard to substance use–related measures, the groups did not differ on alcohol use and related problems (*Z* =  − 0.80, *p* = 0.42; AUDIT), number of cigarette smokers (*χ*^*2*^(1, *N* = 69) = 0.16, *p* = 0.69), number of cigarettes per day (*t*(23) = 0.008, *p* = 0.99) or nicotine dependence (*t*(28) = 0.92, *p* = 0.36; FTND), but heavy cannabis users reported higher lifetime other substance use (*Z* =  − 4.48, *p* < 0.001) and higher cannabis use and related problems (*Z* =  − 7.30, *p* < 0.001; CUDIT) than control participants. Additional sample characteristics are reported in Table 1.

### N-back performance

**Fig. 2:**
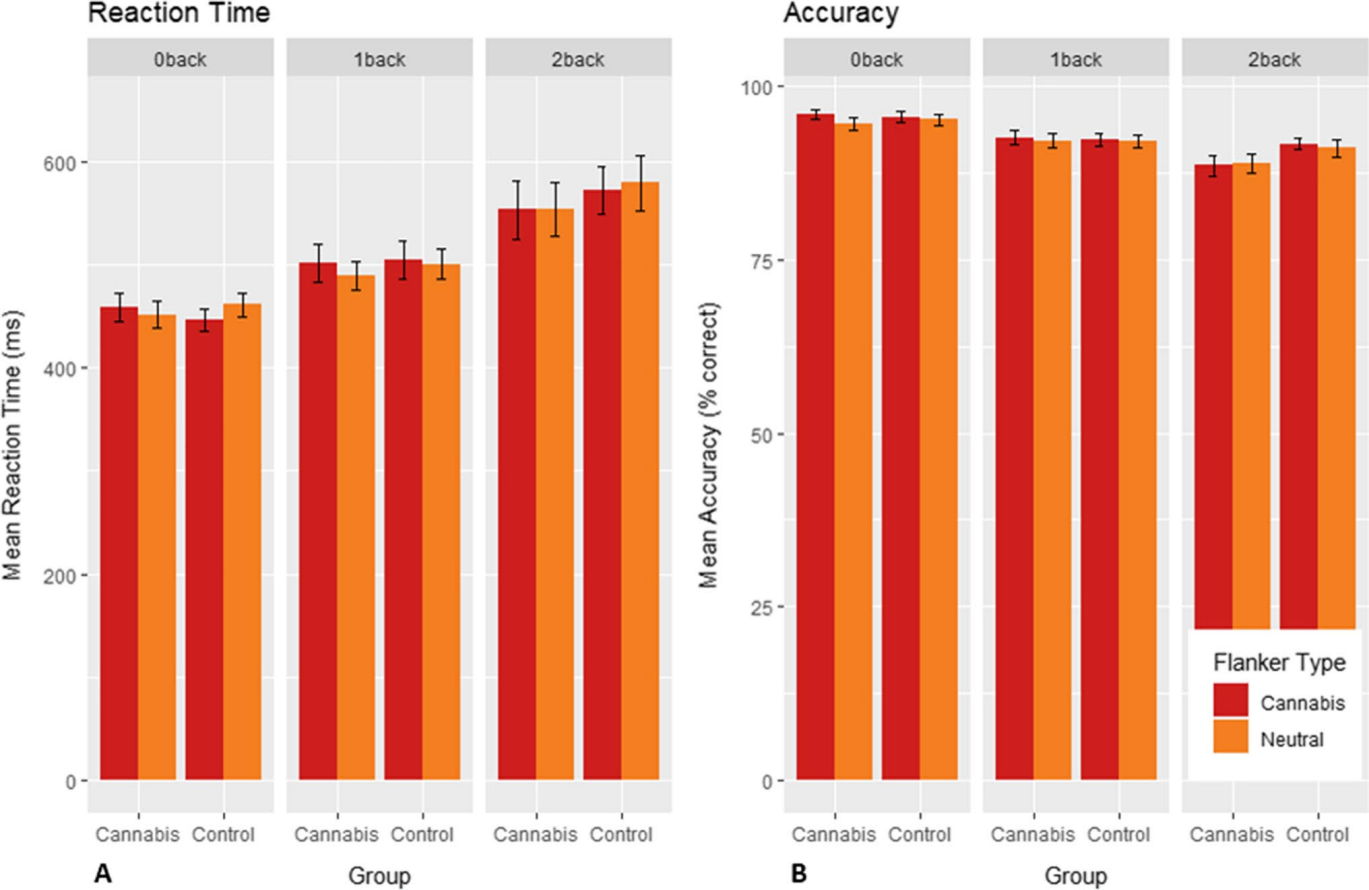
N-back flanker performance. **A** Mean reaction times for 0-back, 1-back and 2-back per flanker type in cannabis users and controls. Reaction time increased with increasing working memory load, independently of group or flanker types (lowest *p*-value < 0.001). **B** Mean accuracy for 0-back, 1-back and 2-back per flanker type in cannabis users and controls. Accuracy decreased with increasing working memory load, independently of group or flanker types (lowest *p*-value = 0.004). Error bars reflect standard error (SE) of the mean

The final model showed that increased WM load negatively affected accuracy (0-back-1-back: *B* =  − 3.00, 95% *CI* =  − 4.43: − 1.57, *p* < 0.001; 0-back-2-back: *B* =  − 5.32, 95% CI =  − 6.75: − 3.88, *p* =  < 0.001; Table 2) as well as reaction time (0-back-1-back: *B* = 44.50, 95% CI = 23.76:65.24, *p* < 0.001; 0-back-2-back: *B* = 109.52, 95% CI = 88.78:130.26, *p* < 0.001; Table 2; Fig. 2). None of the assessed models revealed a significant effect of flanker type, group or any of their interactions on accuracy or reaction time (see Supplementary Table S2 for full model selection).

**Table 2:** Final selected models showing the effect of working memory (WM) load on accuracy and reaction time during the N-back task

Model	Model coefficients					
	Fixed effects					Random effects
Accuracy	B	95% CI (B)	SE (B)	*t*	*p*	*SD*
(Intercept)	92.28	94.03:96.52	0.63	150.83	< 0.001	3.03
WM load: 1-back	− 3.00	− 4.43: − 1.57	0.73	− 4.12	< 0.001	2.95
WM load: 2-back	− 5.32	− 6.75: − 3.88	0.73	− 7.31	< 0.001	
Flanker	-	-	-	-	-	4.04
Reaction time	B	95% CI (B)	S(B)	*t*	*P*	*SD*
(Intercept)	454.36	428.86:479.87	12.98	35.00	< 0.001	88.04
WM load: 1-back	44.50	23.76:65.24	10.53	4.23	< 0.001	53.07
WM load: 2-back	109.52	88.78:130.26	10.53	10.40	< 0.001	
Flanker	-	-	-	-	-	38.66

Mixed model results using random intercept and maximum likelihood estimation. *CI*, confidence interval; *SE*, standard error; *SD*, standard deviation.

### fMRI analysis

**Fig. 3:**
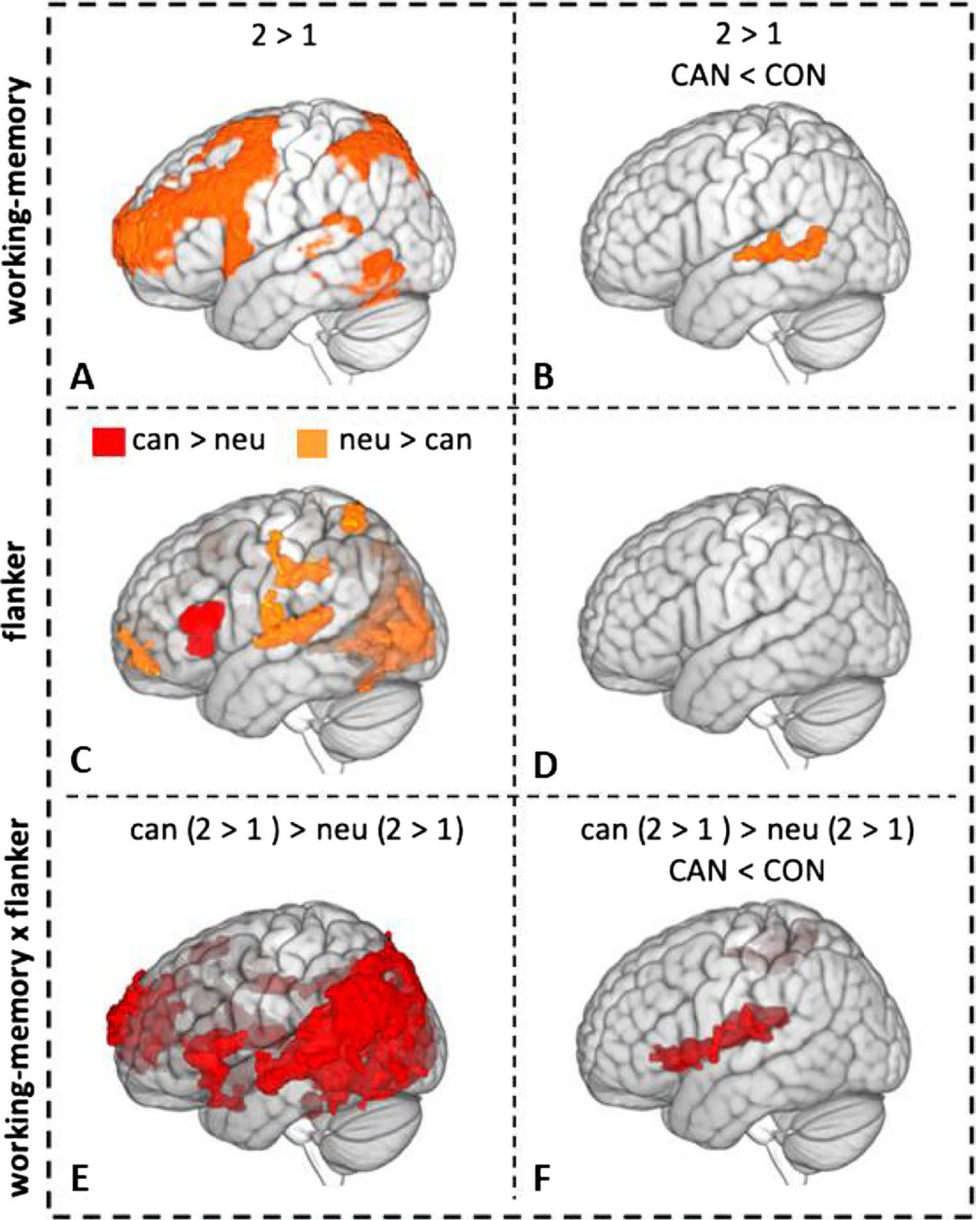
fMRI results. **A** 2 > 1 working memory–related brain activity across groups; **B** group difference (cannabis group < control group) in 2 > 1 working memory–related brain activity; **C** flanker-related activation (cannabis > neutral) and deactivation (neutral > cannabis); **D** no group differences in flanker-related activation; **E** activity for the interaction between working memory load and flanker type; **F** group differences (cannabis group < control group) in activity for the interaction between working memory load and flanker.

**Fig. 4:**
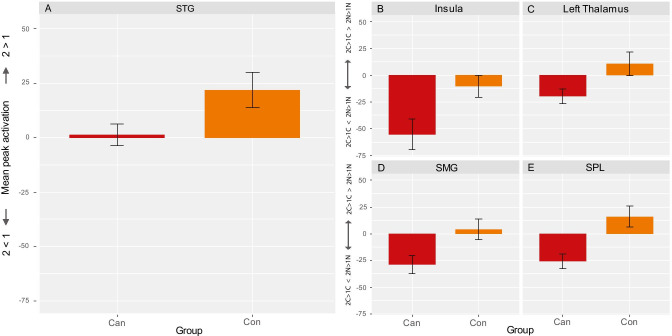
Group differences in mean peak activation in significant clusters found for the WM and interaction contrasts. **A** Group differences in mean working memory–related (2 > 1) peak activation (unitless beta-estimates) of the superior temporal gyrus (STG) (MNI coordinates: *X* =  − 64, *Y* =  − 28, *Z* = 4). Group differences in mean interaction ((2c > 1c) > (2n > 1n)) related activation (unitless beta-estimates) of the **B** insula (MNI coordinates: *X* =  − 40, *Y* = 18, *Z* =  − 2), **C** left thalamus (MNI coordinates: *X* =  − 10, *Y* =  − 22, *Z* = 16), **D** supramarginal gyrus (SMG; MNI coordinates: *X* = 44, *Y* =  − 38, *Z* = 48), **E** superior parietal lobe (SPL; MNI coordinates: *X* = 18, *Y* =  − 50, *Z* = 60). Figure 4A reflects differences in activation for 1-back and 2-back trials, with positive values being indicative of higher mean peak activation for 2-back trials compared to 1-back trials (2 > 1) and negative values reflecting the reverse (1 > 2); Fig. 4B–E reflect working memory–related activity (2 > 1) where positive values reflect relatively higher working memory–related activity for cannabis flankers (2C > 1C > 2 N > 1 N), and negative values reflect the reverse (2C > 1C < 2 N > 1 N). Error bars reflect standard error (SE) of the mean

Increased WM load resulted in increased activity in a widespread network of frontoparietal regions known to be involved in WM performance (Fig. 3A; full overview in Supplementary Table S2) (Owen et al. 2005). In a group comparison, controls showed significantly higher WM-related activation in the superior temporal gyrus (STG), middle temporal gyrus (MTG) and angular gyrus (Fig. 3B*;* Table 3). Post hoc analysis of extracted mean peak activity showed that these differences emerged from controls having increased WM-related brain activity in the STG when presented with more difficult WM trials, while there was close to no difference in activity between 2-back and 1-back trials in heavy cannabis users (Fig. 4A).

**Table 3:** Group differences in activation for the flanker, working memory and interaction contrast

					MNI coordinates				
	Comparison	Cluster size (voxels)	Brain regions	Hemisphere	***X***	***Y***	***Z***	Zmax	***f***^2^
Flanker effect									
c > n	Can > Con	ns	ns	ns	ns	ns	ns	ns	ns
c > n	Con > Can	ns	ns	ns	ns	ns	ns	ns	ns
WM Effect									
2 > 1	Can > Con	ns	ns	ns	ns	ns	ns	ns	ns
2 > 1	Con > Can	420	STG	Left	− 64	− 28	4	3.35	0.20
			MTG	Left	− 60	− 52	6	3.20	0.18
			Angular gyrus	Left	− 54	− 54	14	2.92	0.15
Flanker × WM interaction effect									
(2c > 1c) > (2n > 1n)	Can > Con	ns	ns	ns	ns	ns	ns	ns	ns
(2c > 1c) > (2n > 1n)	Con > Can	1301	Thalamus	Left	− 12	− 20	16	3.35	0.20
			Operculum	Left	− 48	− 22	14	3.33	0.20
			Insula	Left	− 40	8	4	3.26	0.19
		731	SPL	Right	18	− 50	60	3.82	0.28
			SMG	Right	44	− 38	48	3.56	0.23
			PCG	Right	46	− 26	46	3.21	0.18

Regardless of group or WM load, flanker-related activity was higher for neutral flankers in a widespread number of areas, while activity was higher for cannabis flankers in the inferior frontal gyrus (IFG) only (Fig. 3C; full overview in Supplementary Table S2). No group difference in flanker-related brain activity was found.

When looking at the interaction between WM load and flanker type, controls showed higher activation in the thalamus, operculum, insula, superior parietal lobe (SPL), supramarginal gyrus (SMG) as well as the postcentral gyrus (Fig. 3D; Table 3). Exploratory analyses of extracted mean peak activity from clusters significant for the interaction effect showed that heavy cannabis users have lower WM-related brain activity in these areas when presented with cannabis flankers compared to neutral flankers (Fig. 4). The control group shows a similar pattern in the insula (Fig. 4B), although less pronounced. However, WM-related brain activity in the left thalamus (Fig. 4C), SMG (Fig. 4D) and SPL (Fig. 4E) is higher in controls when presented with cannabis flankers compared to neutral flankers.

*MNI*, Montreal Neurological Institute; MNI coordinates and *Z*-scores of separate local maxima for each cluster (whole-brain cluster-corrected at *p* < 0.05, Z > 2.3); *c*, cannabis flanker; *n*, neutral flanker; *Can*, cannabis group; *Con*, control group; *1*, 1-back; *2*, 2-back; *STG*, superior temporal gyrus; *MTG*, middle temporal gyrus; *SPL*, superior parietal lobe; *SMG*, supramarginal gyrus; *PCG*, postcentral gyrus; effect size: *f*^2^ ≥ 0.02 = small, *f*^2^ ≥ 0.15 = medium, *f*^2^ ≥ 0.35 = large.

Further exploratory analyses revealed that cannabis use (grams per week) and dependence (DSM-5 symptom count) were not predictive of the observed differences in brain activity (smallest *p*-value = 0.51). With regard to performance (accuracy and reaction time), WM load–related activity (2–1) in the STG could not be predicted by WM load–related performance (2–1) in the cannabis group (accuracy: *β* = 0.38, *t*(31) = 0.60, *p* = 0.55; reaction time: *β* =  − 0.02, *t*(31) = 0.50, *p* = 0.62) nor control group (accuracy: *β* = 0.38, *t*(23) = 0.26, *p* = 0.80; reaction time: *β* = 0.03, *t*(23) = 0.29, *p* = 0.78).

**Fig. 5:**
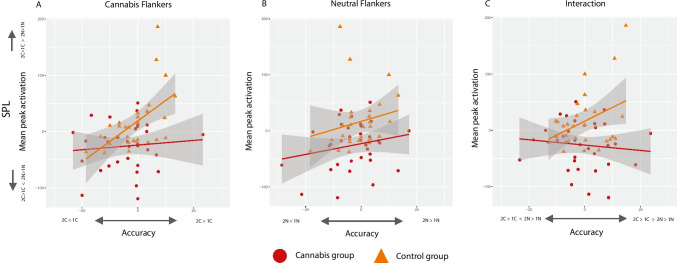
Association between mean peak activation of the SPL and accuracy. **A** Mean peak activation (unitless beta-estimates) in the SPL for the interaction contrast plotted against the performance on cannabis flanker trials; **B** mean peak activation (unitless estimates) in the SPL for the interaction contrast plotted against the performance on neutral flanker trials; **C** Mean peak activation (unitless estimates) in the SPL for the interaction contrast plotted against the performance on cannabis flanker trials minus the performance on neutral flanker trials (interaction); grey area reflects standard error (SE) of the mean

For the interaction effect (2C – 1C)—(2 N – 1 N), performance was not predictive of activity in the insula, thalamus, SMG and SPL in the cannabis group (smallest uncorrected *p*-value accuracy = 0.39 (SMG); smallest uncorrected *p*-value reaction time = 0.50 (insula)). Similarly, no significant results were found for the reaction time data in controls (smallest uncorrected *p*-value reaction time = 0.07 (SPL)). This was different for the accuracy data in the control group, where interaction-related accuracy ((2C – 1C)—(2 N – 1 N)) was predictive of interaction-related brain activity ((2C – 1C)—(2 N – 1 N)) in the SPL (*β* = 2.46, *t*(26) = 2.08, uncorrected *p*-value = 0.048; Fig. 5C). Further visual inspection of the data (Fig. 5) revealed that this effect was guided by a positive association between relative increased activity for the WM effect in cannabis flankers (compared to neutral flankers; y-axis Fig. 5A) and a relatively higher performance for 2-back trials with cannabis flankers (compared to 1-back trials with cannabis flankers; x-axis Fig. 5A). This effect was not observed in the cannabis group or for the neutral flanker trials (Fig. 5B). While multiple comparison correction was not performed due to the explorative nature of these analyses, it must be noted that the significant association between interaction-related accuracy and interaction-related brain activity in the SPL (uncorrected *p*-value = 0.048) is not significant when the Bonferroni correction is applied (corrected *p*-value = 0.192).

## Discussion

We aimed to elucidate the role of a distracting cannabis context in WM load–dependent performance as well as the related brain activity in heavy cannabis users. In contrast to our expectations, the presence of cannabis flankers did not reduce WM load–dependent performance in cannabis users. However, fMRI results showed that in heavy cannabis users compared to controls, the presence of cannabis flankers related to less WM load–related activity than neutral flankers did in multiple regions including the insula, thalamus, SPL and SMG. These results suggest that the presence of cannabis words affects brain activity underlying attention reliant cognitive processes like WM in cannabis users, and the brain areas involved highlight the potential role of saliency (Peters et al. 2016), attention (Vandenberghe et al. 2012), somatosensory processing (Saadon-Grosman et al. 2020) and sensorimotor integration (Wolpert et al. 1998) herein.

While a different activation pattern emerged for cannabis flankers compared to neutral flankers, no group differences were found. Nevertheless, flanker type seems to affect brain activity at a higher WM load only, with reduced activity for cannabis versus neutral flankers in the left insula, left thalamus, right SMG and right SPL in cannabis users, but not controls. Previous studies in non-cannabis users showed that increased cognitive effort for emotional stimuli during a WM task can result in reduced activity in emotion-related areas, while having no effect on WM performance (Erk et al. 2007; Grimm et al. 2012). This is in line with the observed reduced activity in the insula and thalamus, areas implicated in SUDs through craving and salience attribution (Garavan 2010; Huang et al. 2018), in response to stimuli with a higher emotional load. The cannabis group also showed reduced WM load–related activity for cannabis flankers compared to neutral flankers in the right SPL and right SMG. This could point towards a cannabis-flanker distraction effect shifting away resources from the SMG and the SPL. The SMG has been implicated in remembering serial order during memory tasks (Guidali et al. 2019) and word processing (Stoeckel et al. 2009), while the SPL is often involved in attentional processes (Shapiro and Hillstrom 2002) and thereby also in WM performance (Koenigs et al. 2009). Exploratory post hoc analyses indicated that the group differences in brain activity could not be explained by behavioural performance on the n-back flanker task.

The cannabis flanker words included in our N-back flanker task have been shown to induce an attentional bias in heavy cannabis users that was stronger in more severe cannabis users (Cousijn et al. 2013b). In contrast to these results, cannabis use and CUD symptom severity did not relate to any of the observed flanker effects on WM load–related brain activity. It is possible that the apparent cannabis flanker distraction effect under high WM load does not directly relate to use or problem severity or that the limited variability in use patterns prevented us from finding an association. Alternatively, the cannabis stimuli may have been of limited salience to the present users, reducing engagement with the stimuli, or processing of the words was limited due to task speed. Future paradigms should explore how flanker modality and relative salience (e.g. picture stimuli or multimodal stimuli) affect performance and related activity in groups with more variable cannabis use, including more severe clinical populations.

The adapted N-back flanker task showed similar behavioural results to previous fMRI studies using the letter N-back (Cousijn et al. 2014b, a; Hatchard et al. 2020). Performance was found to be WM load dependent, with accuracy going down and reaction times going up with increasing difficulty, accompanied by increasing WM load–related activity in frontoparietal regions. In contrast to our previous study in heavy cannabis users (Cousijn et al. 2014a), we found higher WM load–related activation in the left STG, MTG and angular gyrus in controls compared to cannabis users. Compared to our previous study, a clear strength of the current study is the close matching of cannabis users and controls on depression, anxiety, alcohol use and cigarette use. These confounding factors may have masked group differences in our previous study. The STG, MTG and angular gyrus are primarily found to be involved in word processing (Kuchinke et al. 2005; Diaz and McCarthy 2009), but the STG has also been implicated in attentional processes (Shapiro and Hillstrom 2002). Exploratory analysis of mean peak activation shows that activity in these regions increased with increased WM load in controls only, a difference that could not be explained by high activity for low WM load in cannabis users. Increased involvement of language processing specific areas might not be surprising as the primary alteration made to the N-back task is adding words as emotional distractors, but the underlying cause for group differences remain entirely speculative. Moreover, our observation of increased WM-related activity in the left STG in cannabis users contradicts several earlier studies that found the exact opposite in the left (Hatchard et al. 2020) and right STG (Kanayama et al. 2004; Smith et al. 2010). The recent study by Hatchard et al. (Hatchard et al. 2020) suggests left STG activity is related to semantic processing during the letter N-back but finds increased activity in the cannabis group rather than the control group. Using different types of WM tasks and relatively small samples, Kanayama et al. (Kanayama et al. 2004) and Smith et al. (Smith et al. 2010) interpreted the observed increases in right STG activity as compensatory activity in cannabis users. Future research should assess how different types of flankers (e.g. words or pictures) and relevance of word stimuli for task performance (e.g. task-irrelevant or task-relevant words) affect task-related brain activity to clarify these apparent contradictions.

Besides the closely matched cannabis users and controls, a clear strength of this study is the addition of distracting cannabis and neutral words to an established task to create a novel N-back flanker task. This allowed us to gain important new insights into the effect of a distracting cannabis context on the neurocognitive mechanisms underlying cognitive control–related processes in heavy cannabis users. Nevertheless, some limitations should be considered. First, the relatively high levels of accuracy indicate a ceiling effect, and future studies are encouraged to incorporate higher WM load (e.g. 3-back trials). Second, groups were not matched on other illicit drug use, potentially confounding the current results. However, total lifetime use in the cannabis group was minimal (median = 12), and exclusion of subjects testing positive on other illicit drugs make it unlikely that (sub-)acute effects of these drugs affected the results. Third, history of cannabis use was determined through self-reports, and the inclusion of more objective measures of cannabis use may gain better insights into associations between brain functionality and cannabis exposure. Similarly, we did not include an objective measure to verify participant adherence to the 24-h cannabis abstinence before the session. While future studies should aim to include more objective verification methods, the lack of a group difference in reaction time and performance on the N-back task indicate that it is unlikely our results are the result of intoxication effects in the cannabis group (Hartman and Huestis 2013). Furthermore, the cross-sectional nature and sample size of our study prevents us from drawing conclusions about causality and the detection of small effects. Our sample size is relatively large compared to existing WM studies in cannabis users (Kanayama et al. 2004; Hatchard et al. 2020), highlighting the general need for larger longitudinal neuroimaging studies and replication studies (Poldrack et al. 2017). Finally, future studies are warranted to assess the replicability of this novel paradigm.

In conclusion, the presence of distracting cannabis-related words reduced WM load–related brain activity in cannabis users compared to controls in various brain areas implicated in saliency, attention, somatosensory processing and sensorimotor integration. This implies that heavy cannabis users process cannabis-related cues differently and that cannabis cue exposure might interfere with other cognitive processes under cognitively demanding circumstances. Future studies should focus on the role of context in cognitive control– and attention-related processes like WM to further elucidate the potential cognitive impairments in heavy cannabis users and how these relate to loss of control over drug seeking itself.

## Supplementary Information

Below is the link to the electronic supplementary material.

Supplementary file1 (PDF 299 KB)
