# Supplementary material for: Context dependent differences in working memory related brain activity in heavy cannabis users
Source: Psychopharmacology (Berl). 2021 Aug 27;239(5):1373–85. doi: 10.1007/s00213-021-05956-y (PMC9110519; doi:10.1007/s00213-021-05956-y)
Supplement: Supplementary file 1 — Supplementary file1 (PDF 299 KB) [file 213_2021_5956_MOESM1_ESM.pdf]

Context Dependent Differences in Working Memory Related Brain Activity in Heavy Cannabis Users

Emese Kroon[1,2], Lauren Kuhns[1,2], Janna Cousijn[1,2,3]

[1]Neuroscience of Addiction (NofA) Lab, Department of Psychology, University of Amsterdam, Amsterdam, The Netherlands

[2]The Amsterdam Brain and Cognition Center (ABC), University of Amsterdam, Amsterdam, The Netherlands

[3]Department of Psychology, Education & Child Studies, Erasmus University Rotterdam, Rotterdam, The Netherlands

*Correspondence: Emese Kroon, e.kroon@uva.nl, P.O. box 15916, 1001 NK Amsterdam, The Netherlands

Table S1 Overview of model selection to assess accuracy and reaction time during the N-back task as a function of working memory (WM)-load, group and flanker type

Model		Model coefficients						Model comparison		
		Fixed effects					Random effects			
**Accuracy**		***B***	**95% *CI* (*B*)**	***SE* (B)**	***t***	***p***	***SD***	**AIC**	***χ2***	***p***
0	(Intercept)	92.50	91.58 : 93.43	.47	196.64	<.001	2.61	2646.391	-	-
	WM-load	-	-	-	-	-	3.98			
	Flanker Type	-	-	-	-	-	3.99			
1	*(Intercept)*	*92.28*	*94.03 : 96.52*	*.63*	*150.83*	*<.001*	*3.03*	*2604.738*	*45.62*	*<.001*
	WM-load: 1-back	*-3.00*	*-4.43 : -1.57*	*.73*	*-4.12*	*<.001*	*2.95*			
	WM-load: 2-back	*-5.32*	*-6.75 : -3.88*	*.73*	*-7.31*	*<.001*				
	Flanker Type	-	-	-	-	-	*4.04*			
2	(Intercept)	95.50	94.19 : 96.81	.67	143.01	<.001	3.03	2605.676	1.06	.30
	WM-load: 1-back	-3.00	-4.43 : -1.57	.73	-4.12	<.001	2.96			
	WM-load: 2-back	-5.31	-6.75 : -3.88	.73	-7.30	<.001				
	Flanker Type: neutral	-.44	-1.28 : 0.40	.43	-1.03	0.31	4.03			
3	(Intercept)	95.06	94.48 : 96.63	.80	118.27	<.001	3.00	2606.725	0.95	.33
	WM-load: 1-back	-3.00	-4.43 : -1.57	.73	-4.11	<.001	2.96			
	WM-load: 2-back	-5.32	-6.75 : -3.88	.73	-7.29	<.001				
	Flanker Type: neutral	-0.44	-1.28 : 0.40	.43	-1.03	.31	4.03			
	Group: control	0.91	-0.95 : 2.78	.94	0.97	.33	-			
4	(Intercept)	95.79	93.90 : 97.85	1.02	94.39	<.001	3.03	2614.568	6.16	.52
	WM-load: 1-back	-3.34	-5.76 : -0.92	1.24	-2.69	0.008	2.86			
	WM-load: 2-back	-7.35	-9.77 : -4.93	1.24	-5.93	<.001				
	Flanker type: neutral	-1.35	-3.36 : 0.66	1.03	-1.31	.19	4.02			
	Group: control	-0.34	-3.23 : 2.54	1.47	-0.23	.82	-			
	WM-load: 1-back * Flanker Type: neutral	0.96	-1.88 : 3.81	1.46	0.66	.51	-			
	WM-load: 2-back * Flanker Type: neutral	1.60	-1.24 : 4.45	1.46	1.10	.27	-			
	WM-load: 1-back * Group: control	0.10	-3.40 : 3.59	1.79	0.05	.96	-			
	WM-load: 2-back * Group: control	3.51	0.01 : 3.91	1.79	1.96	.05	-			
	Flanker type: neutral * Group: control	1.00	-1.90 : 3.91	1.50	0.67	.50	-			
	WM-load: 1-back * Flanker Type: neutral * Group: control	-0.79	-4.90 : 3.32	2.12	-0.37	.71	-			
	WM-load: 2-back * Flanker Type: neutral * Group: control	-1.88	-5.99 : 2.23	2.12	-0.89	.38	-			

Mixed model results using random intercept and maximum likelihood estimation; CI: Confidence Interval; SE: Standard Error; SD: Standard deviation; AIC: Akaike information criterion.
Note: final models as presented in the manuscript are presented in italic.

**Table S1 Continued**										
**Model**		**Model coefficients**						**Model comparison**		
		**Fixed effects**					**Random effects**			
**Reaction Time**		**B**	**95% CI (B)**	**SE (B)**	***t***	***p***	***SD***	**AIC**	***χ2***	***P***
0	(Intercept)	505.70	483.16 : 528.24	11.45	44.18	<.001	82.11			
	WM-load	-	-	-	-	-	76.49	4819.618	-	-
	Flanker Type	-	-	-	-	-	37.75			
1	*(Intercept)*	*454.36*	*428.86 : 479.87*	*12.98*	*35.00*	*<.001*	*88.05*			
	WM-load: 1-back	*44.50*	*23.76 : 65.24*	*10.53*	*4.23*	*<.001*				
	WM-load: 2-back	*109.52*	*88.78 : 130.26*	*10.53*	*10.40*	*<.001*	*53.07*	*4742.542*	*81.08*	*<.001*
	Flanker Type	-	-	-	-	-	*38.66*			
2	(Intercept)	454.63	428.77 : 480.49	13.18	34.49	<.001	88.05			
	WM-load: 1-back	44.50	23.76 : 76.24	10.54	4.22	<.001				
	WM-load: 2-back	109.52	88.78 : 130.26	10.54	10.39	<.001	53.07	4744.526	0.02	.90
	Flanker Type: neutral	-0.54	-9.10 : 8.03	4.37	-0.12	.90	38.66			
3	(Intercept)	450.19	416.54 : 483.84	17.17	26.22	<.001	87.93			
	WM-load: 1-back	44.50	23.76 : 65.24	10.55	4.22	<.001	53.07			
	WM-load: 2-back	109.52	88.78 : 130.26	10.55	10.38	<.001		4746.362	0.16	.68
	Flanker Type: neutral	-0.54	-9.10 : 8.03	4.37	-0.12	.90	38.66			
	Group: control	9.29	-36.34 : 54.91	23.00	0.40	.69	-			
4	(Intercept)	458.64	421.96 : 495.33	18.88	24.29	<.001	87.99			
	WM-load: 1-back	42.73	10.71 : 74.75	16.43	2.60	.01	52.94			
	WM-load: 2-back	94.52	62.50 : 126.55	16.43	5.75	<.001				
	Flanker type: neutral	-7.39	-27.73 : 12.95	10.47	-0.71	.48	38.23			
	Group: control	-12.18	-65.88 : 41.51	27.30	-0.45	.66	-			
	WM-load: 1-back * Flanker Type: neutral	-4.62	-33.39 : 24.14	14.80	-0.31	.76	-			
	WM-load: 2-back * Flanker Type: neutral	7.99	-20.78 : 36.75	14.80	0.54	.59	-	4754.888	5.47	.60
	WM-load: 1-back * Group: control	15.73	-30.57 : 62.03	23.76	0.66	.51	-			
	WM-load: 2-back * Group: control	30.70	-15.60 : 77.00	23.76	1.29	.20	-			
	Flanker type: neutral * Group: control	21.91	-7.50 : 51.33	15.14	1.45	.15	-			
	WM-load: 1-back * Flanker Type: neutral * Group: control	-14.40	-55.99 : 27.20	21.41	-0.67	.50	-			
	WM-load: 2-back * Flanker Type: neutral * Group: control	-15.39	-56.99 : 26.20	21.41	-0.72	.47	-			

Mixed model results using random intercept and maximum likelihood estimation; CI: Confidence Interval; SE: Standard Error; SD: Standard deviation; AIC: Akaike information criterion.
Note: final models as presented in the manuscript are presented in italic.

Table S2 Activation overview for the flanker and working memory (WM) contrasts

				MNI coordinates			
	**Cluster size (voxels)**	**Brain regions**	**Hemisphere**	**X**	**Y**	**Z**	**Zmax**
**Flanker**							
**c > n**	469	IFG	Left	-50	26	12	4.17
**n > c**	44257	Intracalcerine cortex	Left	-18	-86	4	5.46
		Frontal pole	Right	36	58	-2	5.40
		Lingual gyrus	Right	10	-84	-4	5.36
		Lingual gyrus	Left	-6	-84	-6	5.34
		Occipital Pole	Left	-10	-94	2	5.21
	1363	Frontal pole	Left	-30	58	-10	4.57
**WM**							
**2 > 1**	38581	MFG	Left	-30	4	58	7.13
		Insula	Right	30	20	8	6.24
		SFG/Paracingulate gyrus	Left	-8	16	48	6.08
	13625	Precuneus	Left	-6	-68	50	6.37
		Lateral occipital	Left	-30	-70	34	6.36
		SPL	Left	-32	-50	42	5.89
		SMG	Left	-38	-48	40	5.86
**1 > 2**	10178	MFG/Paracingulate gyrus	Left	-8	40	-10	5.66
	1160	Central operculum	Right	42	-14	16	4.17
	1139	Cingulate gyrus	Left	-2	-48	30	5.62
	606	Central operculum	Left	-44	0	14	4.11

MNI = Montreal Neurological Institute; MNI coordinates and Z-scores of separate local maxima for each cluster (whole-brain cluster-corrected at $p < 0.05$, $Z > 2.3$); c = cannabis flanker, n = neutral flanker; 1 = 1-back, 2 = 2-back; IFG = Inferior Frontal Gyrus, MFG = Medial Frontal Gyrus, SFG = Superior Frontal Gyrus, SPL = Superior Parietal Lobe, SMG = Supramarginal Gyrus.
